# Colloidal Synthesis, Characterization, and Photoconductivity of Quasi-Layered CuCrS_2_ Nanosheets

**DOI:** 10.3390/nano12234164

**Published:** 2022-11-24

**Authors:** Jose J. Sanchez Rodriguez, Andrea N. Nunez Leon, Jabeen Abbasi, Pravin S. Shinde, Igor Fedin, Arunava Gupta

**Affiliations:** Department of Chemistry and Biochemistry, The University of Alabama, Shelby Hall, Tuscaloosa, AL 35487, USA

**Keywords:** photoconductivity, colloidal synthesis, semiconductors, heat-up, bandgap, absorber, TMDC, layered, nanomaterials

## Abstract

The current need to accelerate the adoption of photovoltaic (PV) systems has increased the need to explore new nanomaterials that can harvest and convert solar energy into electricity. Transition metal dichalcogenides (TMDCs) are good candidates because of their tunable physical and chemical properties. CuCrS_2_ has shown good electrical and thermoelectrical properties; however, its optical and photoconductivity properties remain unexplored. In this study, we synthesized CuCrS_2_ nanosheets with average dimensions of 43.6 ± 6.7 nm in length and 25.6 ± 4.1 nm in width using a heat-up synthesis approach and fabricated films by the spray-coating method to probe their photoresponse. This method yielded CuCrS_2_ nanosheets with an optical bandgap of ~1.21 eV. The fabricated film had an average thickness of ~570 nm, exhibiting a net current conversion efficiency of ~11.3%. These results demonstrate the potential use of CuCrS_2_ as an absorber layer in solar cells.

## 1. Introduction

In the context of accelerating global warming caused by fossil fuel-based hydrocarbon energy, it is essential to develop alternative ways of energy production that shift away from fossil fuels. Hence, efficient photovoltaic (PV) systems with strong absorber layers are a leading alternative approach to capturing and converting solar energy [1]. The commercially available PV systems are principally composed of crystalline silicon due to its long-term stability and high abundance. It is the gold standard in the industry; however, silicon’s poor light-absorbing properties require thicker layers, leading to higher production costs and making it difficult to fabricate as flexible solar cells. The alternative, thin-film solar cells, are known as the second-generation technology because they offer an opportunity to develop flexible high-specific power photovoltaics due to their high optical absorption coefficients, desirable bandgaps, and self-passivated surfaces [2]. In recent years, 2D transition metal dichalcogenides (TMDCs) have emerged as attractive thin-film solar cell materials because of their unique physical and chemical properties. For example, the bandgap of well-known TMDCs, such as MoSe_2_, WSe_2_, MoS_2_, and WS_2_, changes from indirect to direct when these materials are reduced from multilayers to a monolayer form [3,4,5]. Therefore, some of the novel 2D TMDCs are direct bandgap semiconductors in monolayer form with excellent electronic properties and tunable bandgaps [6,7]. Due to their layered nature, these materials can potentially enable us to fabricate flexible, thinner, more efficient, and versatile solar cells [8]. 2D TMDCs offer excellent electron transport properties and high carrier mobility compared to commercially available absorber materials [9]. Current absorber materials with commercial potential are held back due to their poor stability and short shelf life [10,11]. In this context, quasi-layered CuCrS_2_ appears to be a promising chalcogenide material that absorbs light over a broad range of the visible electromagnetic spectrum and exhibits a good photoconductive response. However, its optical properties have not been thoroughly investigated.

CuCrS_2_ is member of a family of layered compounds ACrX_2_ of the early transition metals with a rhombohedral structure (space group *R*3*m*) [12]. It is a well-known thermoelectric material with a thermal conductivity of 0.53 Wm^−1^K^−1^ that crystallizes in a sandwich-like structure with alternating layers of CrS_2_ perpendicular to the c-axis, linked together by weak van der Waals forces and intercalated by Cu atoms [9,13,14,15,16,17]. The electrical measurements by Negard et al. and Chen et al. suggest a semiconducting behavior of CuCrS_2_ [13,18], and subsequent reports indicate mixed conductor properties with an electrical conductivity of 10^−4^ S cm^−1^ at low temperatures [19,20]. Theoretical and experimental studies suggest that CuCrS_2_ is a p-type material with narrow direct and indirect bandgaps of 1.354 and 0.58 eV, respectively [17,21]. On the contrary, a study on bulk CuCrS_2_ reports conflicting data with an optical bandgap of 2.48 eV [22]. Moreover, photoconductivity studies on nanocrystals of CuCrS_2_ are not available. Hence, there is scope to further investigate this material to gain more convincing insights. Due to its promising optical properties, absorption in the visible range, and suitable direct bandgap, CuCrS_2_ can serve as a potential absorber material in solar cells.

This work examines the optical, electrical, and photoconductive properties of CuCrS_2_ nanosheets. We report the synthesis of phase-pure CuCrS_2_ nanosheets using a heat-up synthesis method. The optical bandgap of the synthesized nanosheets was determined to be 1.21 ± 0.06 eV. For the electrical measurements, the thin films of CuCrS_2_ were fabricated using a nano-ink spray-coating method. The film demonstrated a significant photoresponse characteristic with an ~11.3% conversion efficiency, making CuCrS_2_ nanosheets attractive as an absorber layer in solar cell devices.

## 2. Materials and Methods

### 2.1. Synthesis and Characterization of Cu Precursor and CuCrS_2_ Nanosheets

#### 2.1.1. Materials

Sodium diethyldithiocarbamate (Na(DDTC) hydrate), copper (II) nitrate trihydrate (Cu(NO_3_)_2_·3H_2_O), chromium (III) chloride anhydrous, and analytical-grade hexane and ethanol were obtained from Sigma-Aldrich (MilliporeSigma, Burlington, MA, USA). All solids used for the synthesis of the Cu precursor were ground using a mortar and pestle and dried at atmospheric pressure and 180 °C for 18 h prior to use. Dichloromethane was purchased from Supelco (Supelco Inc., Bellefonte, PA, USA) and used without further purification. Oleylamine (OLA) distilled (min 99%) was obtained from Silver Fern (Silver Fern Chemical Inc., Seattle, WA, USA) and 1-dodecanethiol (1-DDT, 98%) was obtained from Alfa Aesar (Alfa Aesar, Ward Hill, MA, USA). These were used as received without any further purification.

#### 2.1.2. Synthesis of Cu Precursor

In a typical reaction, copper (II) nitrate trihydrate (Cu(NO_3_)_2_·3H_2_O) (0.11 g; 0.562 mmol) was suspended in 10 mL of dichloromethane. Sodium diethyldithiocarbamate (0.18 g; 1.06 mmol) was dissolved in 15 mL of dichloromethane. The copper (II) nitrate suspension was added to the sodium diethyldithiocarbamate solution under strong stirring. At contact, the sheer yellow solution turned black. After 30 min, the solvent was removed under vacuum, leaving a black precipitate. The obtained precursor, copper (Diethyldithiocarbamate)_2_, was used without further purification. Yield: 0.15g, 73%. ^1^H NMR (CDCl_3_): δ = 3.50–3.46 (q, 8H, N-CH_2_), 1.22–1.19 (t, 12H, -CH_3_). ^13^C NMR (CDCl_3_): δ = 11.07 (-CH_3_), 42.09 (N-CH_2_), 107.61 (CS_2_). MS (ESI): Calc. for C_10_H_20_CuN_2_S_4_ ([M]^+^): *m*/*z* 358.98. Found: *m*/*z* 358.80; found for C_10_H_20_CuN_2_S_4_^+^ in positive mode, with characteristic Cu isotope pattern.

#### 2.1.3. Synthesis of CuCrS_2_ Nanosheets

In a typical synthesis of layered CuCrS_2_ nanosheets, 0.25 mmol of the Cu(DDTC)_2_, 0.25 mmol of CrCl_3_, and 2 mL of 1-DDT were added to a three-neck flask connected to a Schlenk line containing 10 mL of OLA. The precursor-containing mixture was then stirred and degassed with N_2_ for 20 min. The solution was heated to 320 °C and maintained at this temperature for 60 min. The resulting dark mixture was then naturally cooled. The solution was split into two vial tubes, and a 1:1 mixture of hexane and ethanol was poured into the solution and centrifugally separated. The precipitate was washed and centrifuged (8000 rpm for 5 min) with hexane and ethanol until the solution became clear.

#### 2.1.4. Characterization Methods

The structural characterization of the precursor was achieved via ^1^H- and ^13^C-NMR, using a BRUKER AVANCE 500 MHz NMR and ESI-MS on a BRUKER HCTultra PTM Discovery System on positive mode (both equipment from Bruker Co., Billerica, MA, USA). Single metallic, dark black, plate-shaped crystals of Cu(DDTC)_2_ were used. A suitable crystal with dimensions of 0.08 × 0.07 × 0.04 mm^3^ was selected and mounted on a Rigaku XtaLAB Synergy R, DW system, and HyPix diffractometer (Rigaku Co., Tokyo, Japan). The single-crystal X-ray diffraction data for the precursor can be found in the Supporting Information in Figure S1 and Table S1. The crystal was kept at a steady temperature of 113(19) K during data collection. The structure was solved with the ShelXT version 2018/2 [23] solution program using dual methods and refined by full-matrix least-squares minimization on F^2^ using version ShelXL 2018/3 [24]. Olex2 1.3 [25] was used as the graphical interface.

The crystallographic analyses of the as-synthesized nanoparticles were performed by X-ray diffraction using a BRUKER D2 diffractometer (Bruker Co., Billerica, MA, USA) with Cu K_α_ (1.5406 Å) radiation. The absorbance spectra were measured using Shimadzu UV-3600i Plus UV-vis-NIR spectrophotometer (Shimadzu Co., Kyoto, Japan), and the optical bandgap was estimated from Tauc plot analysis. Scanning electron microscopy analysis was carried out using an Apreo FE-SEM (Thermo Fisher Scientific Inc., Waltham, MA, USA) to probe the surface morphology and thickness of the sprayed thin films prepared from nanoparticle suspension. The chemical composition was determined using energy-dispersive X-ray analysis (EDX). Transmission electron microscopy (TEM) and high-resolution TEM analyses were performed using an FEI Tecnai 20 (Thermo Fisher Scientific Inc., Waltham, MA, USA) with a 200 kV operating beam. The nanosheets were dispersed in hexane and dropped onto a carbon-coated nickel TEM grid. Current and voltage (I–V) measurements were performed in dark and under one-sun (100 mW cm^−2^, AM 1.5 G) light illumination conditions using a Princeton potentiostat (AMETEK Inc., Berwyn, PA, USA) in the applied potential window of −500 mV and +500 mV.

## 3. Results and Discussion

### 3.1. Copper Precursor Spectral Studies and Characterization

**Figure 1:**
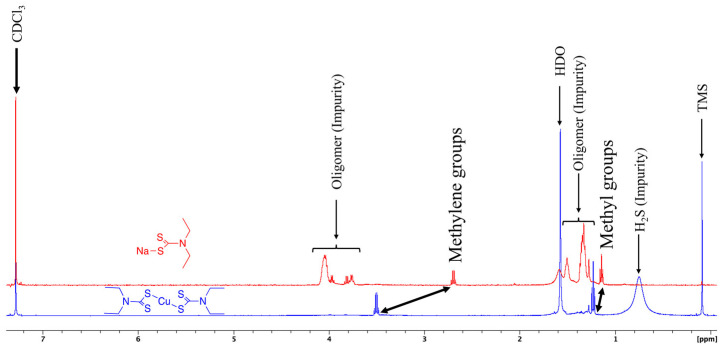
Comparison of ^1^H-NMR of Na(DDTC) (red) and Cu(DDTC)_2_ (blue).

Through the obtained ^1^H-NMR spectra of the ligand and the complex, the formation of the target compound and an improvement in the purity comparing the starting material to the product was verified. The quartet corresponding to the methylene protons in the free ligand was found at 2.7 ppm and shifted to 3.46 and 3.50 ppm in the final product. The quartet was shifted downfield due to the deshielding effect of the neighboring nitrogen. The triplet that corresponded to the methyl groups was found at 1.19 ppm and shifted downfield to 1.22 ppm, as seen in Figure 1. In the ^13^C-NMR spectrum in Figure S2, the methyl groups were found at 11.07 ppm, the methylene carbons at 42.09 ppm, and the diethyldithiocarbamate carbon (NCS_2_) at 107.61 ppm. The mass spectrum in Figure S3 confirmed the expected mass of 359 Da for Cu(DDTC)_2_, including the characteristic Cu isotope pattern.

**Figure 2:**
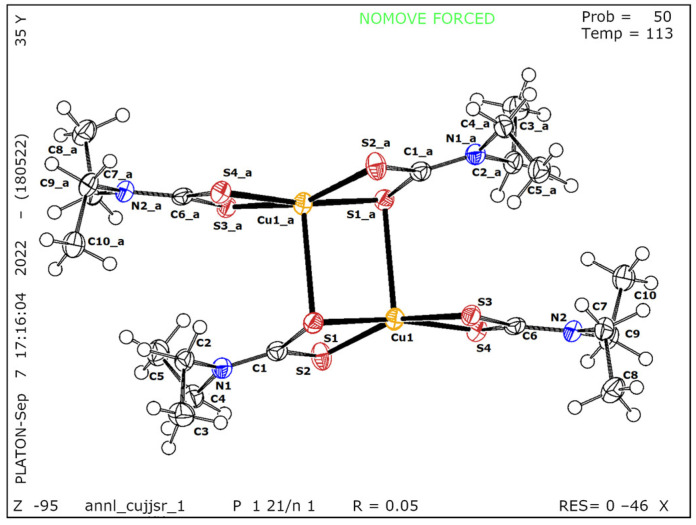
Crystal structure of Cu(DDTC)_2_.

The determined structure (Figure 2) reveals that the complex is built up of centrosymmetric dimeric entities, where the copper (II) ions sit in a distorted square–pyramidal coordination sphere, consistent with the structures previously reported [26,27]. The basal coordination positions are occupied by four sulfur atoms belonging to the two ligand units. The fifth coordination location is formed by linking two monomeric units through a sulfur atom. This sulfur atom then occupies the equatorial site in the coordination polyhedron of the aforementioned copper (II) ion. Therefore, each bridging sulfur simultaneously occupies an equatorial coordination site on one copper (II) ion and an axial site on the other Cu (II).

Crystal Data: C_10_H_20_CuN_2_S_4_, M_r_ = 360.06, monoclinic, P2_1_/n (No. 14), a = 9.6823(2) Å, b = 10.5158(2) Å, c = 15.4388(3) Å, b = 101.484(2)°, a = g = 90°, V = 1540.46(5) Å^3^, T = 113(19) K, Z = 4, Z’ = 1, m (Cu K_a_) = 6.909, and 7090 reflections measured, 2916 unique (R_int_ = 0.0404), which were used in all calculations. The final wR_2_ was 0.1389 (all data), and R_1_ was 0.0509 (I ≥ 2 s (I)).

### 3.2. Nanosheets: Synthesis and Characterization

**Figure 3:**
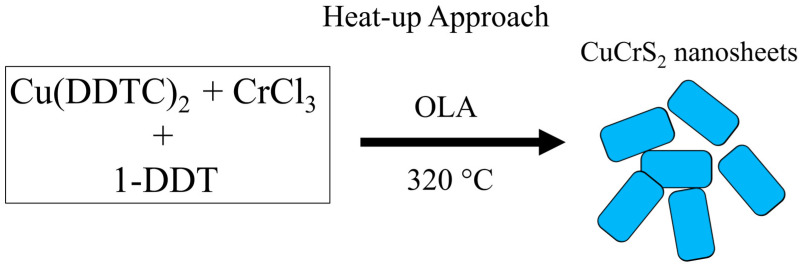
Schematic representation of the heat-up synthesis scheme to obtain CuCrS_2_ nanosheets.

**Figure 4:**
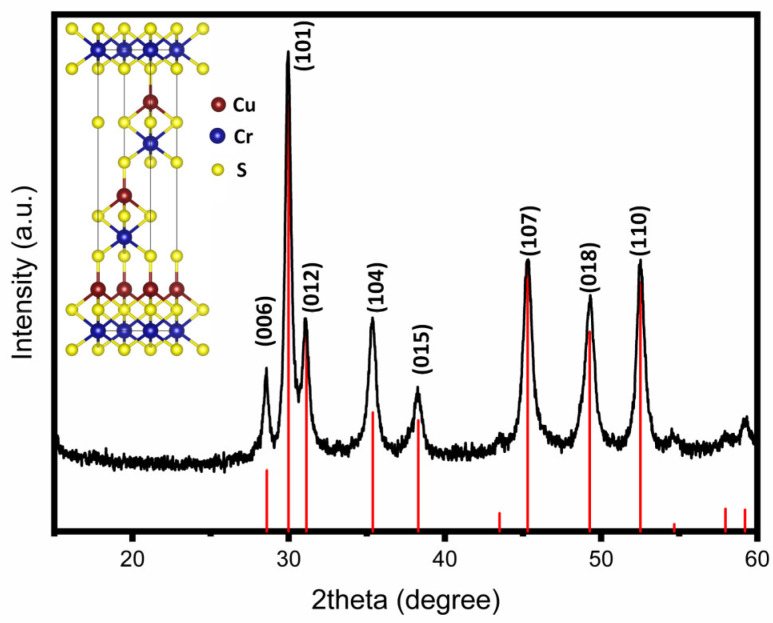
XRD pattern of the colloidal nanosheets obtained after reacting Cu(DDTC)_2_ and CrCl_3_ at 320 °C for 60 min in the presence of 1-DDT and OLA. The vertical red lines represent the standard diffraction data standard (ICDD# 01-079-7417) for CuCrS_2_. The unit cell structure of CuCrS_2_ is shown in the inset.

As shown in Figure 3 a colloidal heat-up strategy was used to synthesize CuCrS_2_ nanosheets. In the heat-up synthesis strategy, all the precursors (Cu(DDTC)_2_, CrCl_3_, and 1-DDT) in a 1:1:8 ratio were dissolved in OLA under a nitrogen environment. The mixture of precursors was heated to 320 °C. During the heat-up process, the precursors decomposed to form monomers. At 270 °C, the XRD pattern (Figure S4) showed the presence of the CuCrS_2_ and Cu_2_S phases. This result suggests that the decomposition of Cu(DDTC)_2_ led to the reduction of Cu^+2^ to the Cu^+1^ state to form Cu_2_S as an intermediate phase, eventually leading to the nucleation of CuCrS_2_. At this temperature, nanohexagons, nanosheets, and impurity phases were spotted simultaneously on TEM images, as seen in Figure S4. The presence of nanohexagons decreased by increasing the temperature to 320 °C. During the heat-up process, the nanohexagons grew in the [100] direction forming nanosheets. It is important to note that different morphologies arose when octadecylamine (ODA) was added as a cosurfactant, as shown in Figure S5. Therefore, the nanosheet morphology can be attributed to using OLA as a surfactant. It is well established that OLA attaches preferentially to different facets of growing nanocrystals [28,29,30,31]. No appreciable morphological changes were detected by varying the time of the reaction. The crystallinity of CuCrS_2_ was determined using X-ray diffraction. As shown in Figure 4, the major diffraction peaks were indexed to the (003), (006), (101), (012), (104), (015), (107), (018), and (110) planes. The observed diffraction peak positions for CuCrS_2_ nanocrystals matched well with the literature and the standard diffraction data (ICDD# 01-079-7417) reported for CuCrS_2_ [12,13,15,17,22,32]. X-ray diffraction analysis indicated that the aforementioned approach produced CuCrS_2_ without any secondary phase. From the diffraction pattern, the lattice parameters were determined to be 3.481 Å (a), 3.481 Å (b), and 18.702 Å (c), consistent with the previously reported data and the reference data [18,33,34,35].

**Figure 5:**
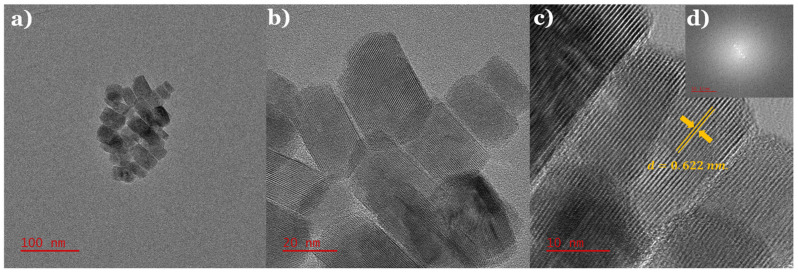
(**a**–**c**) show TEM and HRTEM images of CuCrS_2_ nanosheets with *d*-spacing and inset (**d**) corresponds to its FFT.

The TEM micrographs show that the prescribed mixture of metal precursors, sulfur source, and solvent led to the formation of regular sheet-shaped nanoparticles with a narrow size distribution. The nanosheets had average dimensions of 43.6 ± 6.7 nm in length and 25.6 ± 4.1 nm in width, as seen from the statistical analysis in Figure S6. In conjunction with XRD, the HR-TEM images, as seen in Figure 5b,c, along with the fast Fourier transform (FFT) in Figure 5d, suggest the single crystalline nature of the nanosheets. Moreover, the average particle size of the nanosheets was estimated using the well-known Debye–Scherrer formula, which relates the size of the nanocrystallites in a solid to the broadening of a peak in the diffraction pattern. [36,37]:

(1) $$D=\frac{0.94\lambda }{\beta cos\theta },$$

where *λ* is the wavelength of the X-ray radiation source Cu-Kα (1.5418 A); *β* is the FWHM (full width at half maximum) corresponding to all the peaks on the diffraction pattern; *θ* is the diffraction angle for lattice planes, and *D* is the particle diameter size. The crystallite size was calculated using all the diffraction peaks in the XRD pattern and taking the average of these. The estimated particle size for the nanosheets was 50.8 ± 5.2 nm, which agreed well with the experimental observations (from the TEM study). The indicated lattice fringe spacing of 0.622 ± 0.006 nm corresponded to the *d*-spacing of the (003) plane. Furthermore, the elemental composition analysis from Figure S7 and Table S2 confirmed the expected atomic composition ratio of 1:1:2.

**Figure 6:**
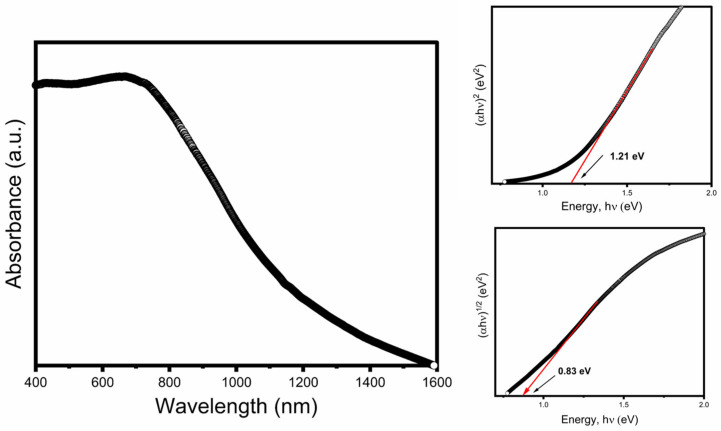
UV-vis absorption spectrum of dispersed CuCrS_2_. The inset shows Tauc plot and extrapolation of the linear portion of the curve on the X-axis at zero absorption to determine the bandgap.

To gain insights into the optical properties of CuCrS_2_, absorption spectroscopy studies were performed. The dispersed nanosheets appeared dark brownish in color, indicating their strong absorption in the visible range of the electromagnetic spectrum. The absorption measurements were obtained from dispersed nanosheets in hexane. Figure 6 shows a typical absorption spectrum of the synthesized CuCrS_2_ nanosheets, exhibiting absorption in the visible range with an optimum absorption of around 700 nm. To estimate the band gap, the Tauc relationship [38,39,40] given below was used:

(2) $${\left(\alpha h\upsilon \right)}^{n}=h\upsilon -E_{g},$$

where *α* is the optical absorption coefficient; *hν* is the photon energy; *n* = 2 or 1/2 for the direct and indirect bandgaps, respectively, and *E_g_* is the energy gap of the material. The direct allowed optical transition was found for CuCrS_2_ with a bandgap energy that can be determined by producing a plot of (αhν)^2^ vs. hν and extrapolating the linear portion of the curve to the horizontal axis of the photon energy at (αhν)^2^ = 0. As shown in the inset of Figure 6, the estimated optical direct and indirect bandgaps of CuCrS_2_ were 1.21 ± 0.06 eV and 0.83 ± 0.03 eV, respectively, which make this material suitable for photovoltaic applications. Nanohexagons of CuCrS_2_, shown in Figure S8, were reported in the literature [35], and upon using the described synthesis approach, we determined the optical direct and indirect bandgaps of the nanohexagons to be 1.29 ± 0.07 eV and 0.80 ± 0.05 eV, respectively, as seen in Figure S9. The slight difference in the values of the bandgaps between the two differently synthesized samples was most likely due to the presence of an impurity phase, as seen in the X-ray diffraction pattern of nanohexagons in Figure S10.

### 3.3. Photoconductivity

**Figure 7:**
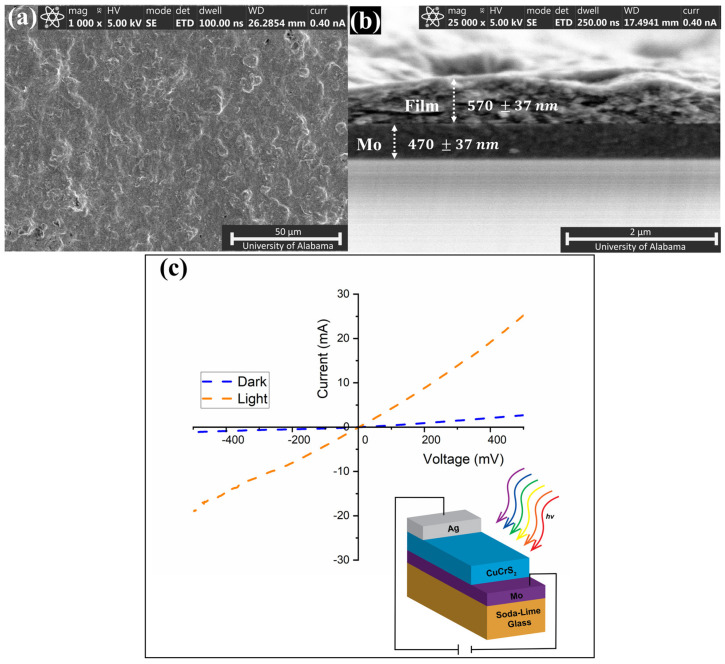
SEM images of (**a**) surface and (**b**) cross-sectional view of 570 nm thick CuCrS_2_ thin film fabricated on Mo-coated glass substrate. (**c**) Current-voltage (I-V) characteristics of CuCrS_2_ thin film structure under white light illumination at 100 mW cm^−^^2^ with an inset showing a schematic of the fabricated structure for I-V measurements.

Semiconducting materials should have an appropriate photoresponse to be suitable for use in solar cell applications. The photoconductivity was determined by analyzing the current–voltage (I–V) characteristics of CuCrS_2_. To study the photoconductivity of CuCrS_2_ nanosheets, the material was incorporated as an absorber layer in a photovoltaic structure composed of soda–lime glass/Mo/CuCrS_2_/Ag, as shown in the inset of Figure 7. The CuCrS_2_ thin films were prepared using a previously reported nano-ink approach [41,42,43]. A “nano-ink” stabilizing solution was prepared by resuspending ~120 mg of CuCrS_2_ in 25 mL of hexane. A few drops of OLA were added to stabilize the suspension. The nano-ink solution was sprayed on top of a molybdenum-coated glass substrate using a commercial spray gun to produce a thin film. The thin film was then annealed in a nitrogen environment at 375 °C for 1 h. Upon further examination, the film had a continuous surface and a thickness of 570 ± 37 nm, as seen in Figure 7a,b. An EDX analysis for the fabricated thin film confirmed the atomic ratio of 1:1:2, as seen in Figure S11 and Table S3.

The conductivity response of the annealed CuCrS_2_ film was measured between the bottom molybdenum layer and top silver electrodes under dark and white light illumination at 100 mWcm^−2^ using a Xenon lamp under air at room temperature. The photocurrent and responsivity of CuCrS_2_ film were calculated from the I–V curve and are summarized in Table 1. The responsivity was calculated using Equation (3) [44]:

(3) $$R_{\lambda }=\frac{\left(I_{L}-I_{D}\right)}{P_{\lambda }\times A},$$

where *R_λ_* is the responsivity (the photocurrent generated per unit power of the incident light used on the CuCrS_2_ film of the effective area (1.09 cm^−2^); *P_λ_* is the intensity of illumination (100 mWcm^−2^); *A* is the effective area of the CuCrS_2_ film, and *I_L_* and *I_D_* are currents generated under illumination and dark, respectively.

The photocurrent of the effective illuminated area of CuCrS_2_ film is shown in Figure 7c and indicates a noticeable increase in photocurrent under illumination. The measured photocurrent was found to be 22.52 mA, and the responsivity was 0.21 A W^−1^. The current conversion efficiency with respect to the dark current was calculated using Equation (4):

(4) $$\mathrm{Efficiency}\;\left(\%\right)=\frac{P_{out}}{P_{in}}\times 100,$$

where *P_in_* is the maximum power generated by the film, and *P_out_* is the intensity of illumination.

The current conversion efficiency of the film was determined to be ~11.3% with respect to the dark current. Our results are comparable to Cu_2_CoSnS_4_ and Cu_2_Cd_x_Zn_1–x_SnS_4_ films for which similar responsivity and current efficiency values have been reported [44,45,46]. The spray-coating system does produce films with a relatively large surface roughness. Nonetheless, the roughness of the fabricated CuCrS_2_ thin film did not influence its photoconductivity, as shown in Figure S12 and Table S4. The respectable photoresponse suggests that CuCrS_2_ can potentially serve as an absorber layer in thin film solar cells.

## 4. Conclusions

In summary, we successfully synthesized and determined the crystal structure of a copper (diethyldithiocarbamate)_2_ precursor and synthesized phase-pure CuCrS_2_ nanoparticles with nanosheet morphology. The synthesis involved the heat-up of a mixture containing the metal precursors, sulfur source, and oleylamine solvent as a capping agent. The structure, phase, morphology, and purity of the synthesized nanosheets were determined by XRD, SEM, and TEM techniques. The nanosheets’ optical direct and indirect bandgaps were determined to be 1.21 ± 0.06 eV and 0.83 ± 0.03 eV from the UV-vis absorption spectra. The fabricated CuCrS_2_ thin film showed good photoresponse characteristics in the current-voltage (I-V) measurements with an ~11.3% current conversion efficiency with respect to the dark measurements. This study demonstrates that CuCrS_2_ nanosheets have potential for their utilization in thin-film solar cell devices.

## Figures and Tables

**Table 1:** Photocurrent and responsivity of CuCrS_2_ thin film electrode.

Bias Voltage (mV)	I_L_ (mA)	I_D_ (mA)	Photocurrent (mA)	Responsivity (A W^−1^)
500	25.30	2.78	22.52	0.21

## Data Availability

The data presented in this study are available on request from the corresponding author.

## Supplementary Materials

The following supporting information can be downloaded at: https://www.mdpi.com/article/10.3390/nano12234164/s1, Figure S1: Crystal structure of Cu(DDTC)2; Table S1: Crystallographic studies on Cu(DDTC)2; Figure S2: 13C-NMR of C(DDTC)2; Figure S3: Positive mode ESI-MS of copper precursor; Figure S4: X-ray diffraction pattern of reaction product at 270 °C; Figure S5: TEM image of the reaction product at 320 °C using OLA and ODA as surfactants; Figure S6: Statistical size analysis and corresponding TEM images of the CuCrS2 nanosheets; Figure S7: EDX spectrum of CuCrS2 nanosheets; Table S2: Elemental composition of CuCrS2 nanosheets determined by EDX; Figure S8: TEM image of CuCrS2 nanohexagons; Figure S9: (a) UV-vis absorption spectra of CuCrS2 nanohexagons, (b) and (c) shows the Tauc plot and extrapolation of the curve to determine the direct and indirect bandgap energy.; Figure S11: EDX spectrum of the fabricated CuCrS2 thin film; Table S3: Elemental composition from EDX of the fabricated thin film after annealing; Figure S12: (a) Current-voltage (I-V) characteristics of CuCrS2 thin film under white illumination at 100 mW cm^−2^. SEM images of (b) the surface continuity and roughness and (c) a cross-section view of ~400 nm thick CuCrS2 thin film fabricated on Mo-coated glass substrate; Table S4: Photocurrent and responsivity of fabricated CuCrS2 thin film.
