# Supplementary material for: Colloidal Synthesis, Characterization, and Photoconductivity of Quasi-Layered CuCrS2 Nanosheets
Source: Nanomaterials (Basel). 2022 Nov 24;12(23):4164. doi: 10.3390/nano12234164 (PMC9736551; doi:10.3390/nano12234164)
Supplement: Supplementary file 1 [file nanomaterials-12-04164-s001.zip › nanomaterials-2020311-supplementary.pdf]

nanomaterials

Supplementary Material

Colloidal Synthesis, Characterization, and Photoconductivity of Quasi-Layered $CuCrS_2$ Nanosheets

Jose J. Sanchez Rodriguez *, Andrea N. Nunez Leon, Jabeen Abbasi, Pravin S. Shinde, Igor Fedin and Arunava Gupta *

Department of Chemistry and Biochemistry, The University of Alabama, Shelby Hall, Tuscaloosa, AL 35487, USA
* Correspondence: jjsanchezrodriguez@crimson.ua.edu (J.J.S.R.); arunava.gupta@ua.edu (A.G.)

List of Figures

Figure S1. Crystal structure of $Cu(DDTC)_2$. 2

Figure S2. ^{13}C-NMR of $Cu(DDTC)_2$. 4

Figure S3. Positive mode ESI-MS of $Cu(DDTC)_2$. 4

Figure S4. X-ray diffraction pattern of reaction product at 270 °C. The vertical red lines correspond to the standard diffraction data of $CuCrS_2$ (ICDD# 01-079-7417), and the vertical blue lines correspond to the standard diffraction data of Cu_2S (ICDD# 01-073-6145). The peaks marked by an asterisk (*) and plus (+) sign represent the $CuCrS_2$ and Cu_2S phases, respectively. The left TEM image shows the reaction at 270 °C. The right TEM image reveals the nanohexagons, which are present early in the reaction and have lattice fringes that correspond to the (003) plane. 5

Figure S5. TEM image of the reaction product at 320 °C using OLA and ODA as surfactants. 6

Figure S6. Statistical size analysis and corresponding TEM images of the $CuCrS_2$ nanosheets. 6

Figure S7. EDX spectrum of $CuCrS_2$ nanosheets. 7

Figure S8. TEM image of $CuCrS_2$ nanohexagons. 7

Figure S9. a) UV-vis absorption spectra of $CuCrS_2$ nanohexagons, b) and c) shows the Tauc plot and extrapolation of the curve to determine the direct and indirect bandgap energy. Bandgaps energies are determined to be 1.29 ± 0.07 eV and 0.80 ± 0.05 eV, respectively. 8

Figure S10. X-ray diffraction pattern of $CuCrS_2$ nanohexagons. The vertical red lines correspond to the standard diffraction pattern of $CuCrS_2$ (ICDD# 01-079-7417). The peaks marked by an asterisk (*) originate due to the impurity phase (Cu_2S). 9

Figure S11. EDX spectrum of the fabricated $CuCrS_2$ thin film. 10

Figure S12. a) Current-voltage (I-V) characteristics of $CuCrS_2$ thin film under white illumination at 100 mW cm^{-2}. SEM images of b) the surface continuity and roughness and (c) a cross-section view of ~400 nm thick $CuCrS_2$ thin film fabricated on Mo-coated glass substrate. 10

List of Tables

Table S1. Crystallographic studies on $Cu(DDTC)_2$. 2

Table S2. Elemental composition of $CuCrS_2$ nanosheets determined by EDX. 7

Table S3. Elemental composition from EDX of the fabricated thin film after annealing. 10

Table S4. Photocurrent and responsivity of fabricated $CuCrS_2$ thin film. 11

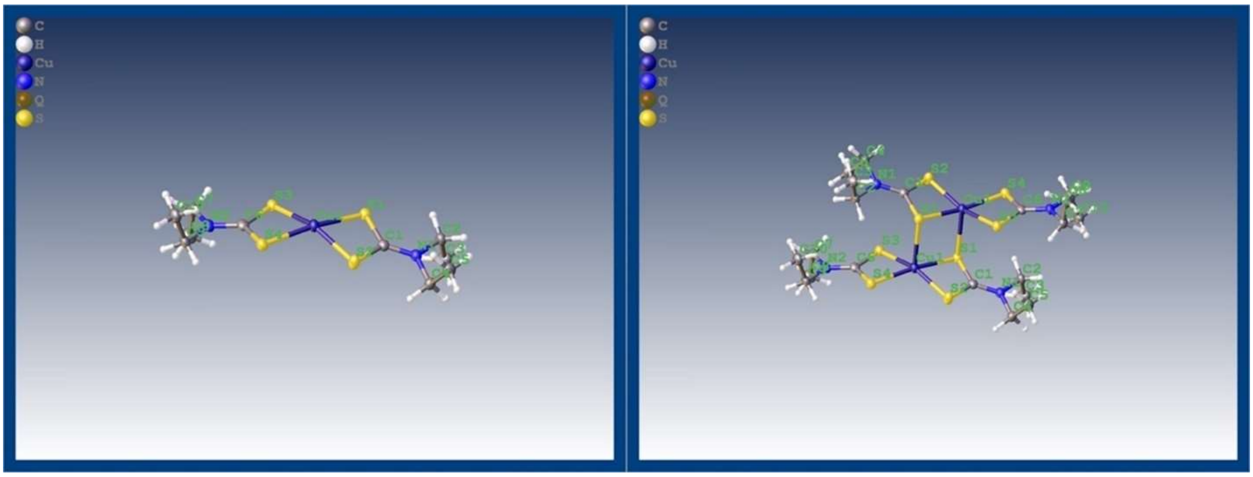

Figure S1. Crystal structure of $Cu(DDTC)_2$.

Table S1. Crystallographic studies on $Cu(DDTC)_2$.

Compound	ANNL_CuJJSR_1
Formula	$C_{10}H_{20}CuN_2S_4$
$D_{calc.}$/ g cm^{-3}	1.552
m/mm^{-1}	6.909
Formula Weight	360.06
Colour	metallic dark black
Shape	plate-shaped

Size/mm^3	0.08×0.07×0.04
T/K	113(19)
Crystal System	monoclinic
Space Group	$P2_1/n$
a/Å	9.6823(2)
b/Å	10.5158(2)
c/Å	15.4388(3)
a/°	90
b/°	101.484(2)
g/°	90
V/Å^3	1540.46(5)
Z	4
Z'	1
Wavelength/Å	1.54184
Radiation type	Cu K_a
Q_{min}/°	4.984
Q_{max}/°	71.196
Measured Refl's.	7090
Indep't Refl's	2916
Refl's I≥2 s(I)	2587
R_{int}	0.0404
Parameters	158
Restraints	0
Largest Peak	1.065
Deepest Hole	-0.962
GooF	1.018
wR_2 (all data)	0.1389
wR_2	0.1351
R_1 (all data)	0.0560
R_1	0.0509

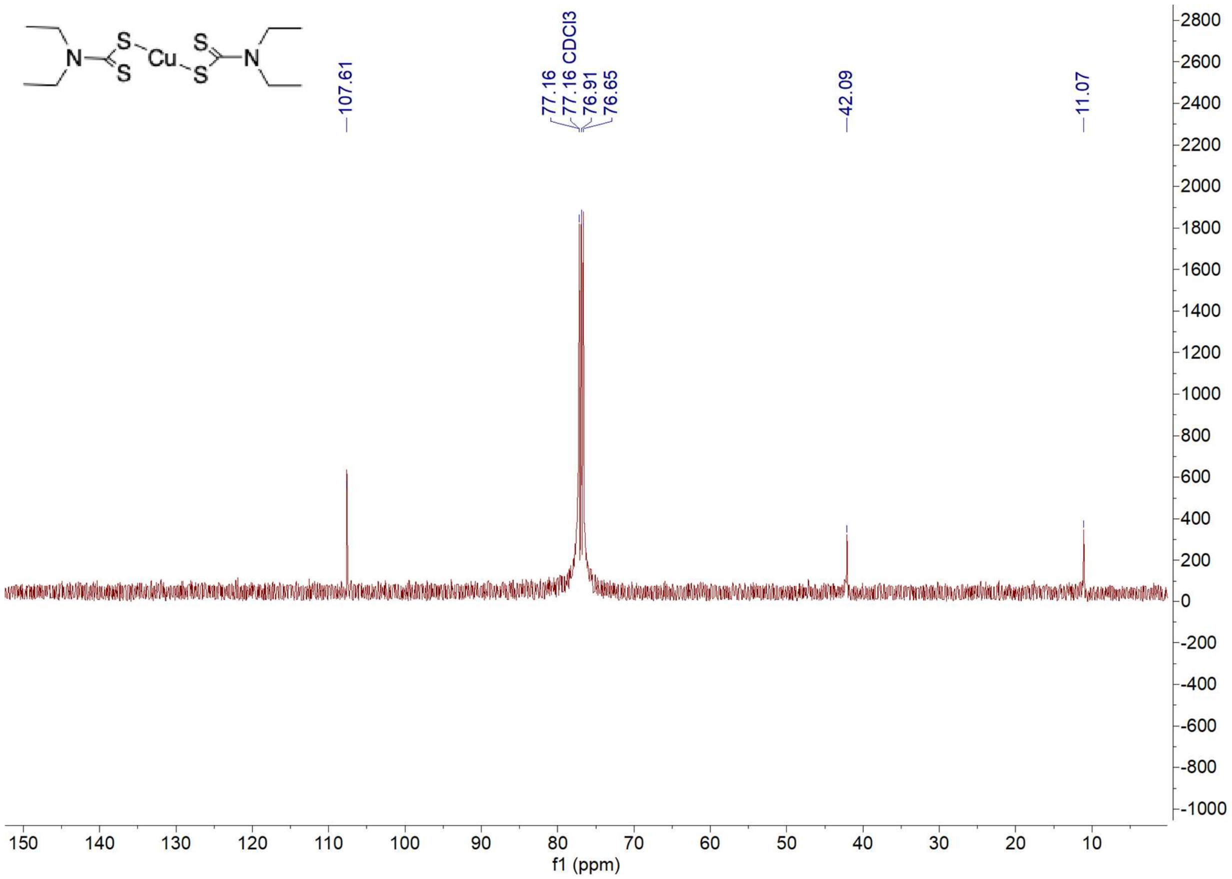

Figure S2. ^{13}C-NMR of Cu(DDTC)2.

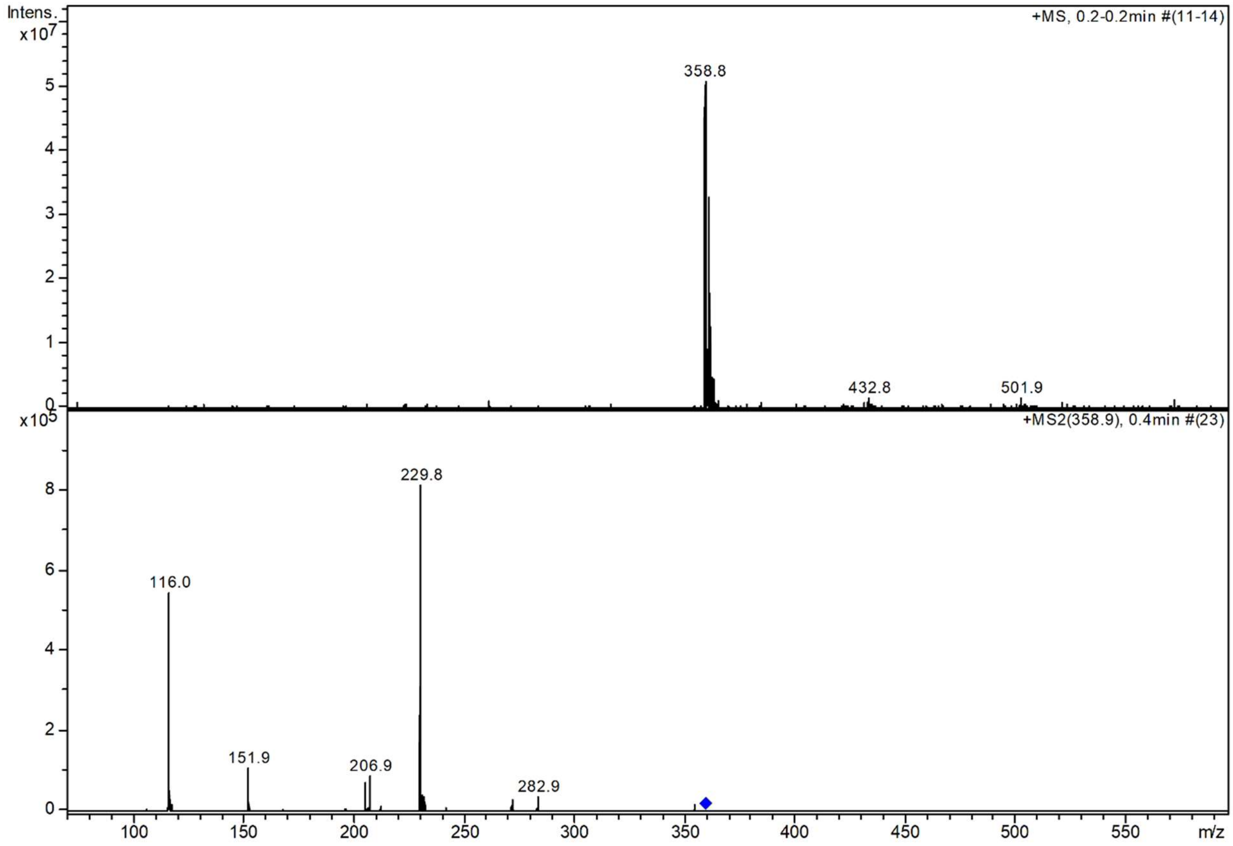

Figure S3. Positive mode ESI-MS of Cu(DDTC)2.

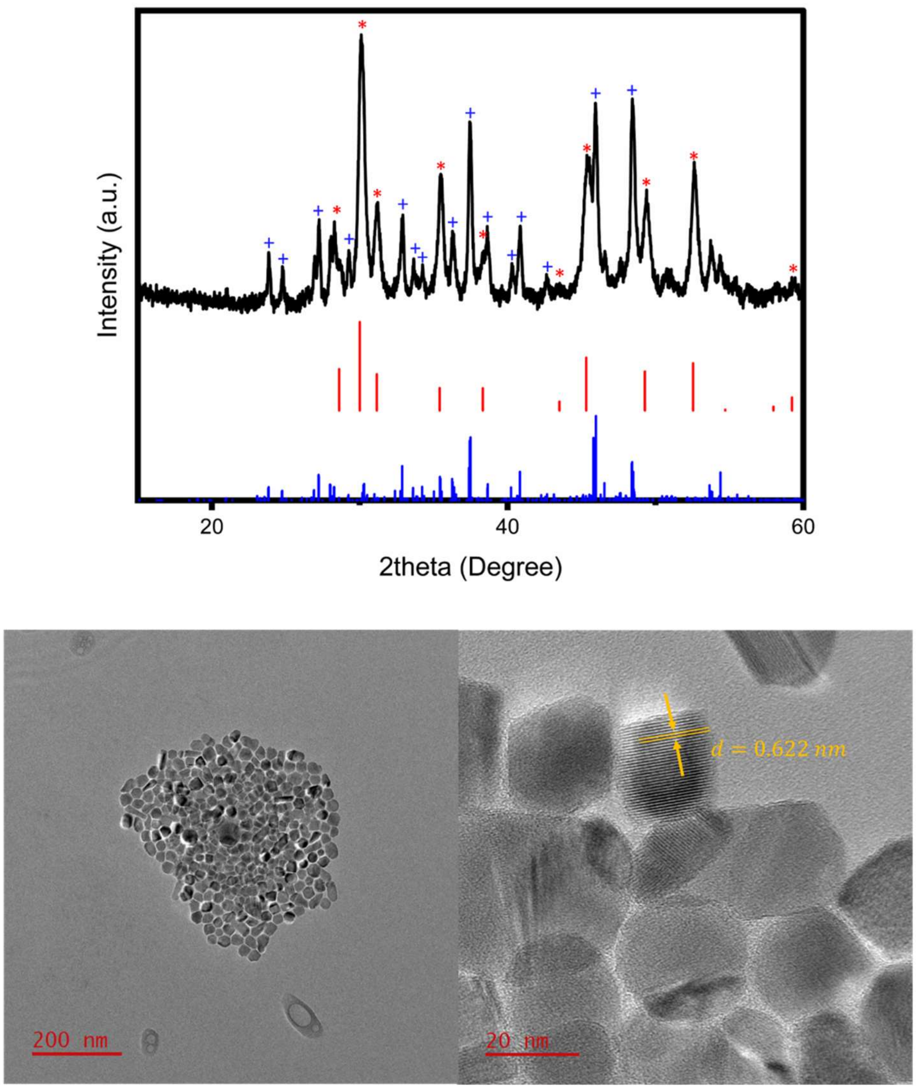

.

Figure S4. X-ray diffraction pattern of reaction product at 270 °C. The vertical red lines correspond to the standard diffraction data of $CuCrS_2$ (ICDD# 01-079-7417), and the vertical blue lines correspond to the standard diffraction data of Cu_2S (ICDD# 01-073-6145). The peaks marked by an asterisk (*) and plus (+) sign represent the $CuCrS_2$ and Cu_2S phases, respectively. The left TEM image shows the reaction at 270 °C. The right TEM image reveals the nanohexagons, which are present early in the reaction and have lattice fringes that correspond to the (003) plane.

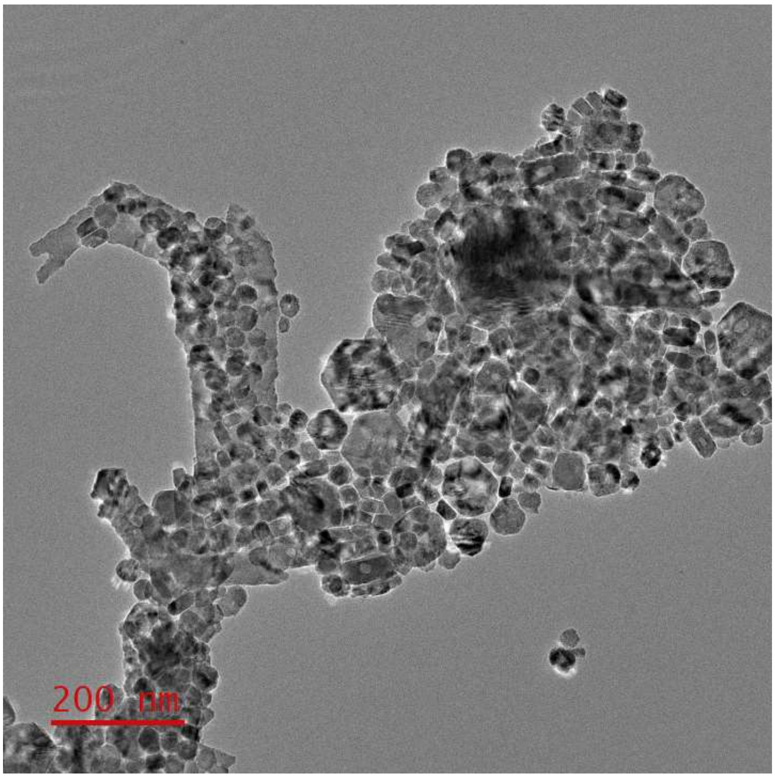

Figure S5. TEM image of the reaction product at 320 °C using OLA and ODA as surfactants.

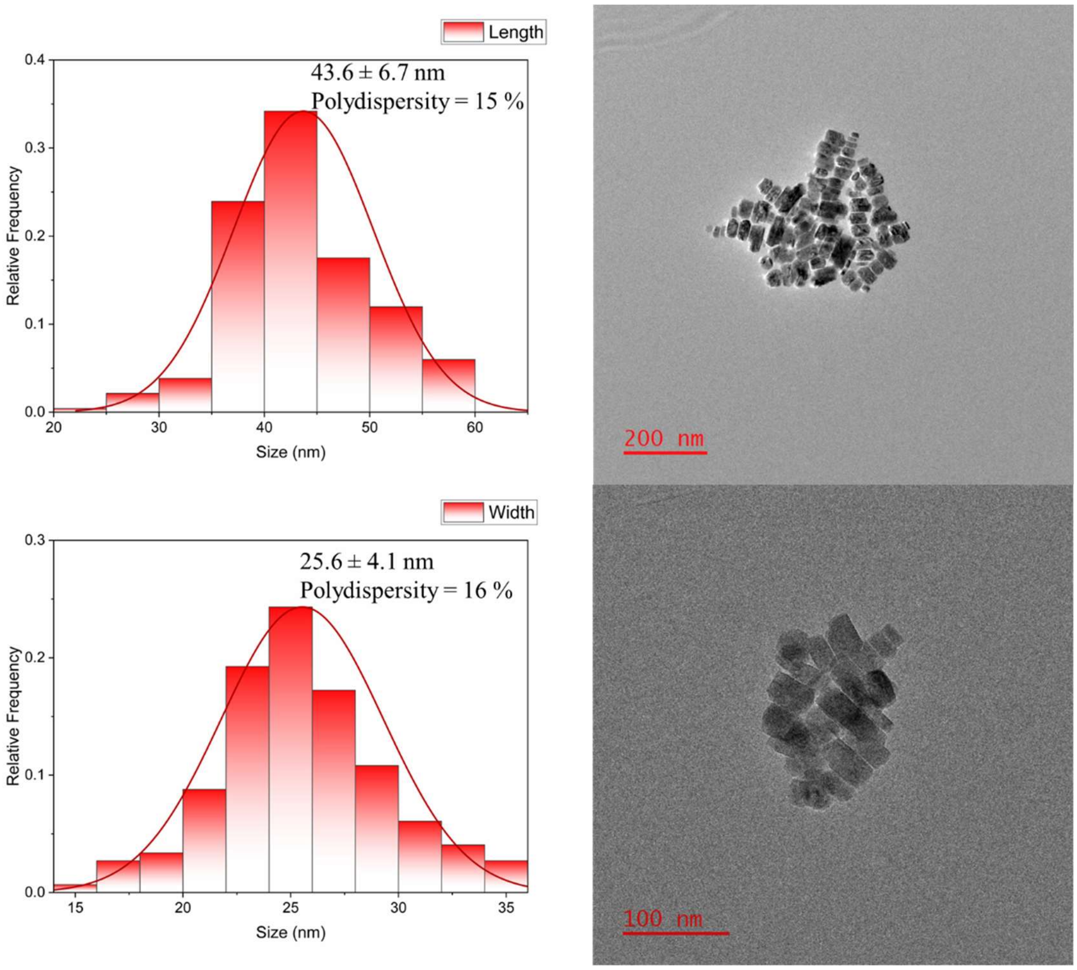

Figure S6. Statistical size analysis and corresponding TEM images of the $CuCrS_2$ nanosheets.

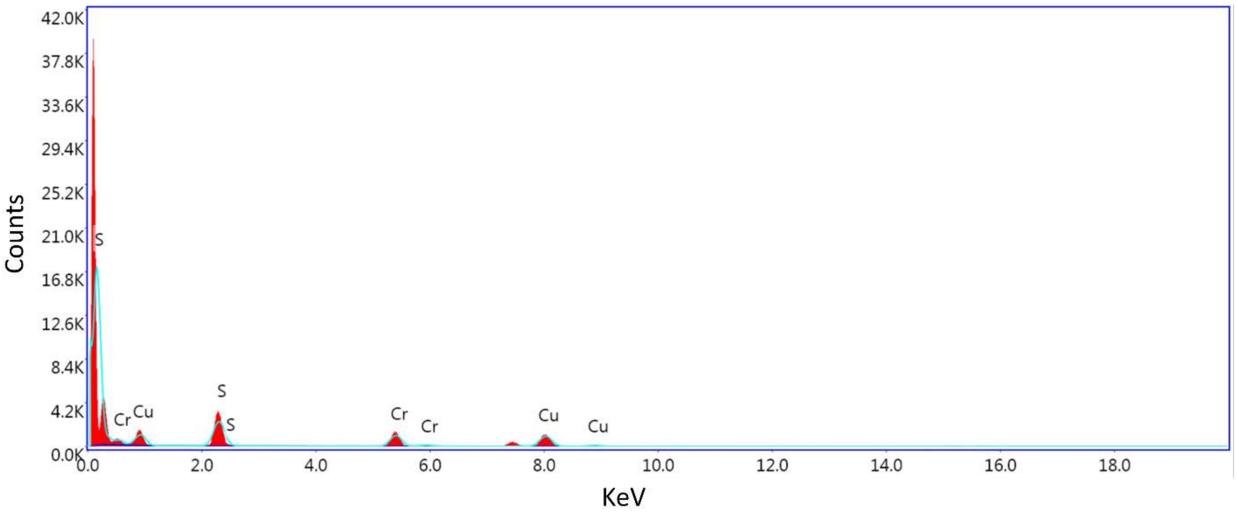

Figure S7. EDX spectrum of $CuCrS_2$ nanosheets.

Table S2. Elemental composition of $CuCrS_2$ nanosheets determined by EDX.

Element	Weight %	Atomic %	Ratio
Cu K	35.15	24.72	1
Cr K	28.22	24.25	1
S K	36.63	51.03	2

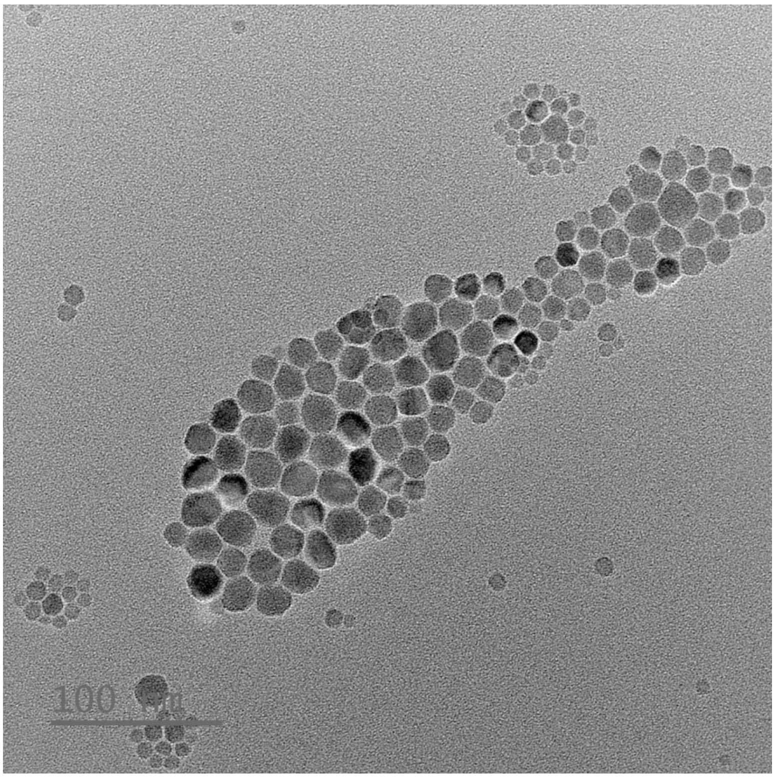

Figure S8. TEM image of $CuCrS_2$ nanohexagons.

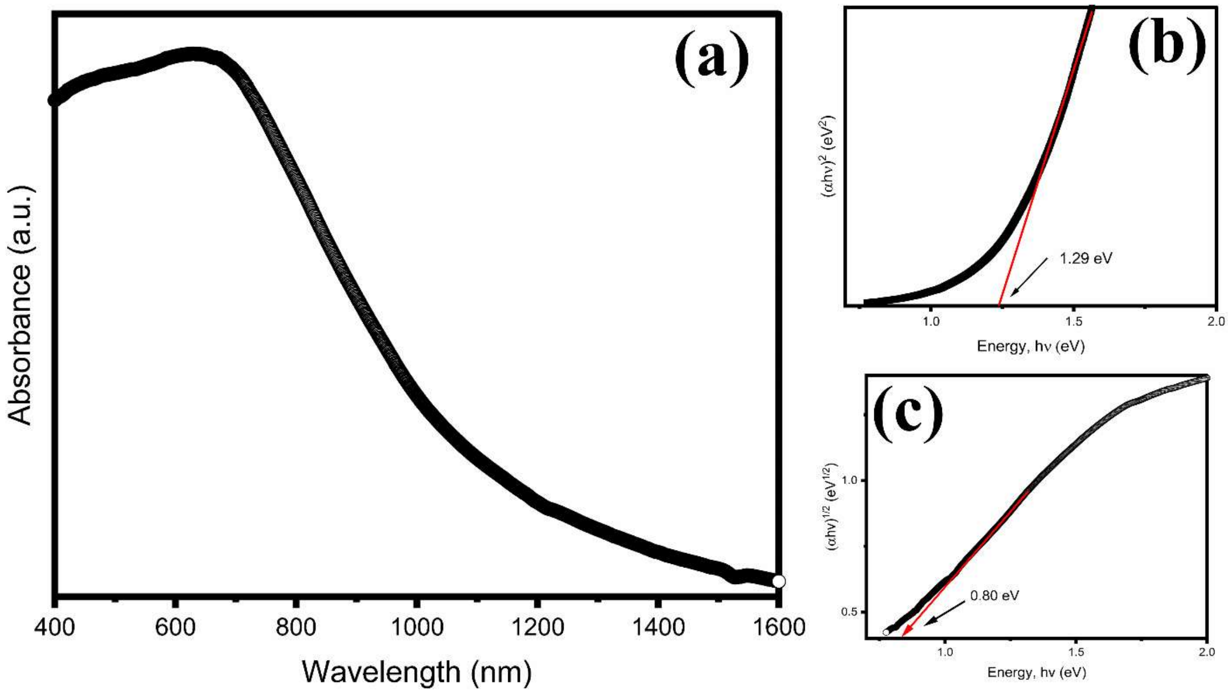

Figure S9. (**a**) UV-vis absorption spectra of $CuCrS_2$ nanohexagons, (**b**) and (**c**) shows the Tauc plot and extrapolation of the curve to determine the direct and indirect bandgap energy. Bandgaps energies are determined to be 1.29 ± 0.07 eV and 0.80 ± 0.05 eV, respectively.

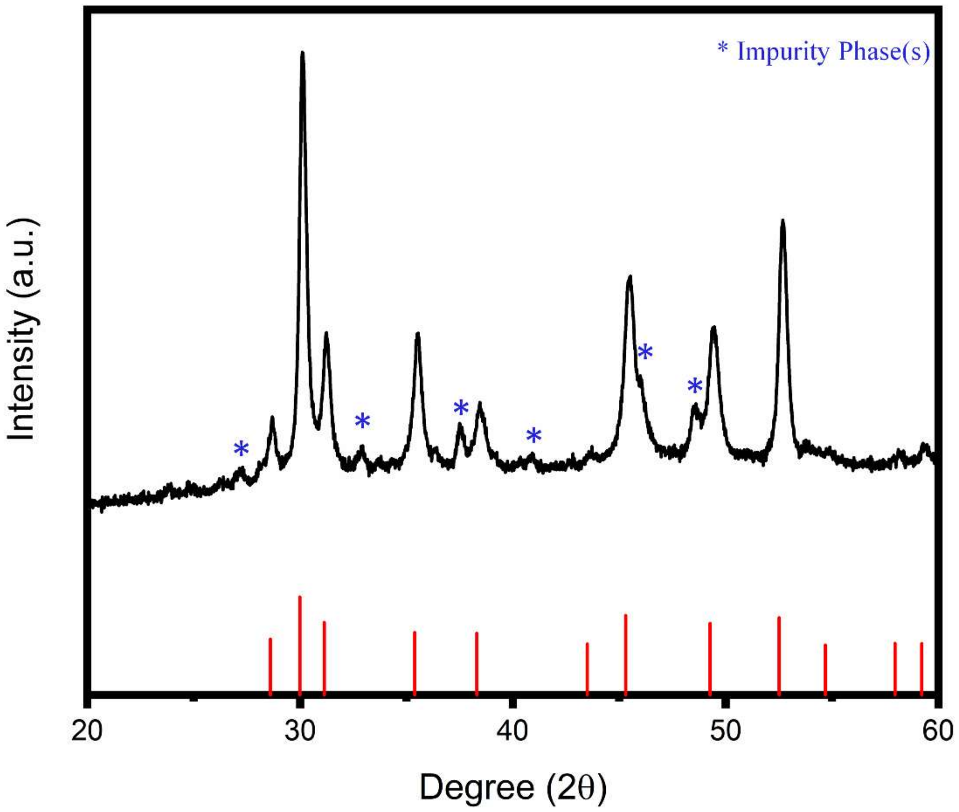

Figure S10. X-ray diffraction pattern of $CuCrS_2$ nanohexagons. The vertical red lines correspond to the standard diffraction pattern of $CuCrS_2$ (ICDD# 01-079-7417). The peaks marked by an asterisk (*) originate due to the impurity phase (Cu_2S).

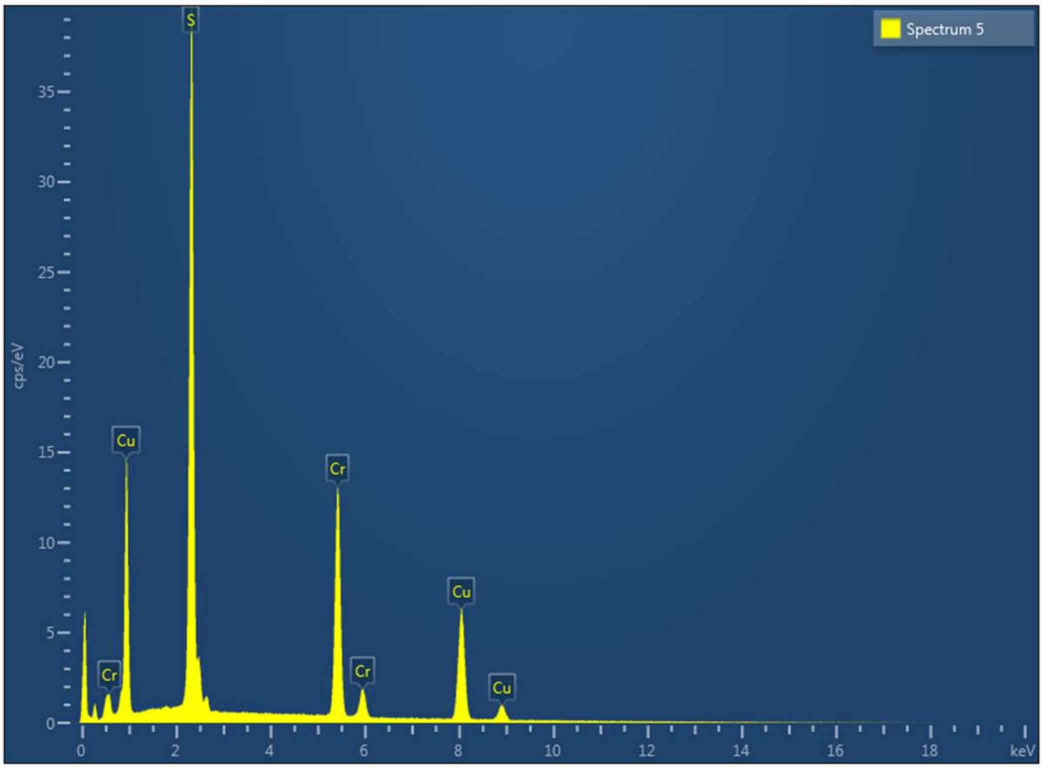

Figure S11. EDX spectrum of the fabricated $CuCrS_2$ thin film.

Table S3. Elemental composition from EDX of the fabricated thin film after annealing.

Element	Atomic %	Ratio
Cu K	25.87	1.04
Cr K	24.91	1.00
S K	49.22	1.98

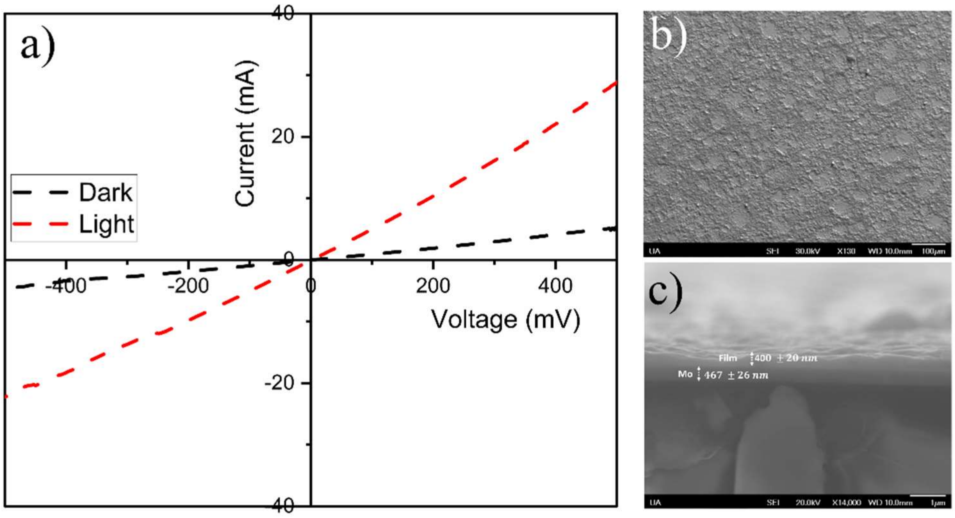

Figure S12. a) Current-voltage (I-V) characteristics of $CuCrS_2$ thin film under white illumination at 100 mW cm^{-2}. SEM images of **b**) the surface continuity and roughness and **c**) a cross-section view of ~ 400 nm thick $CuCrS_2$ thin film fabricated on Mo-coated glass substrate.

Table S4. Photocurrent and responsivity of fabricated $CuCrS_2$ thin film.

Bias voltage (mV)	Effective area (cm^2)	I_L (mA)	I_D (mA)	Photocurrent (mA)	Responsivity ($A\ W^{-1}$)	Efficiency (%)
500	1.12	28.98	4.50	24.48	0.22	10.9

The $CuCrS_2$ thin film fabricated separately for photoconductivity measurement shows relatively less surface roughness and thickness than the original sample. However, the responsivity and the current efficiency with respect to dark current are still comparable, suggesting that the prescribed roughness and thicknesses do not influence photoconductivity. It is important to note that the SEM analysis for this sample was performed using a JEOL 7000 FE SEM at operating voltages of 20 and 30 kV.
